# Unlocking the Key to Accelerating Convergence in the Discrete Velocity Method for Flows in the Near Continuous/Continuous Flow Regimes

**DOI:** 10.3390/e25121609

**Published:** 2023-11-30

**Authors:** Linchang Han, Liming Yang, Zhihui Li, Jie Wu, Yinjie Du, Xiang Shen

**Affiliations:** 1 Department of Aerodynamics, College of Aerospace Engineering, Nanjing University of Aeronautics and Astronautics, Nanjing 210016, Chinawuj@nuaa.edu.cn (J.W.);; 2 State Key Laboratory of Mechanics and Control of Mechanical Structures, Nanjing University of Aeronautics and Astronautics, Nanjing 210016, China; 3 Key Laboratory of Unsteady Aerodynamics and Flow Control, Ministry of Industry and Information Technology, Nanjing 210016, China; 4 National Laboratory for Computational Fluid Dynamics, China Aerodynamics Research and Development Center, Beijing 100191, China; 5 Department of Mechanical and Construction Engineering, Northumbria University, Newcastle upon Tyne NE1 8ST, UK

**Keywords:** Boltzmann-BGK equation, discrete velocity method, convergence acceleration, fully implicit, inner iteration

## Abstract

How to improve the computational efficiency of flow field simulations around irregular objects in near-continuum and continuum flow regimes has always been a challenge in the aerospace re-entry process. The discrete velocity method (DVM) is a commonly used algorithm for the discretized solutions of the Boltzmann-BGK model equation. However, the discretization of both physical and molecular velocity spaces in DVM can result in significant computational costs. This paper focuses on unlocking the key to accelerate the convergence in DVM calculations, thereby reducing the computational burden. Three versions of DVM are investigated: the semi-implicit DVM (DVM-I), fully implicit DVM (DVM-II), and fully implicit DVM with an inner iteration of the macroscopic governing equation (DVM-III). In order to achieve full implicit discretization of the collision term in the Boltzmann-BGK equation, it is necessary to solve the corresponding macroscopic governing equation in DVM-II and DVM-III. In DVM-III, an inner iterative process of the macroscopic governing equation is employed between two adjacent DVM steps, enabling a more accurate prediction of the equilibrium state for the full implicit discretization of the collision term. Fortunately, the computational cost of solving the macroscopic governing equation is significantly lower than that of the Boltzmann-BGK equation. This is primarily due to the smaller number of conservative variables in the macroscopic governing equation compared to the discrete velocity distribution functions in the Boltzmann-BGK equation. Our findings demonstrate that the fully implicit discretization of the collision term in the Boltzmann-BGK equation can accelerate DVM calculations by one order of magnitude in continuum and near-continuum flow regimes. Furthermore, the introduction of the inner iteration of the macroscopic governing equation provides an additional 1–2 orders of magnitude acceleration. Such advancements hold promise in providing a computational approach for simulating flows around irregular objects in near-space environments.

## 1. Introduction

The Boltzmann equation, being free from the limitations of continuum assumption, provides a versatile tool for delineating the molecular transport phenomena that form the bedrock of intricate gas dynamics studies [1,2]. However, the original collision term in the Boltzmann equation involves complex fivefold integration in both molecular velocity space and solid angle, presenting a formidable challenge in solving it. To facilitate practical applications, several simplified collision models have been developed, including the BGK model [3], the Shakov-BGK model [4], and the ES-BGK model [5], among others. These models distill the fundamental and mean characteristics of the original collision integral while providing physical realism [6,7]. Building upon the Boltzmann-BGK equation, numerous numerical algorithms have been devised to solve gas flow problems across all flow regimes [8,9,10,11,12,13]. These algorithms offer convenience and efficiency in simulating complex flows.

The Boltzmann-BGK equation, with its first-order partial derivative, can be easily solved using numerical techniques commonly employed in conventional computational fluid dynamics [14,15]. Within conventional DVM, the convective term is typically evaluated using upwind schemes. These include the third-order upwind scheme [16], the second-order scheme integrated with a slope limiter function [17], the essentially non-oscillatory (ENO) scheme [18], the weighted essentially non-oscillatory (WENO) scheme [19], and other similar methodologies. These approaches involve constructing an initial piecewise constant distribution using the upwind scheme and directly computing the numerical flux at the cell interface, relying on moments of the initial distribution function.

The computation process in the conventional DVM is straightforward, as each discrete distribution function evolves independently. However, this approach introduces numerical dissipation linked to the mesh size, stemming from the omission of collisional effects at the cell interface. Consequently, a mesh size smaller than the mean free path of the molecules is usually required [20]. Furthermore, in the conventional DVM, the time step size is typically limited by the collision time when explicitly discretizing the collision term within the Boltzmann-BGK equation. To overcome this limitation, implicit discretization has been incorporated into the conventional DVM [21,22]. Nevertheless, due to the dependence of the equilibrium state on macroscopic flow variables that are unknown at the new time level, a semi-implicit scheme often becomes necessary for discretizing the collision term. Mieussens pointed out that this semi-implicit discretization may considerably impede the convergence rate, especially in near continuum and continuum flow regimes [23].

Within the realm of DVM, various multiscale approaches have been designed to provide accurate and efficient predictions of flows across the continuum, near-continuum, and rarefied flow regimes. These methods aim to surmount the limitations tied to mesh size and time step size in the conventional DVM by utilizing the multiscale local solution of the Boltzmann-BGK equation for calculating numerical flux. Notable examples encompass the unified gas kinetic scheme (UGKS) [24,25,26] and the discrete unified gas kinetic scheme (DUGKS) [27,28,29]. The UGKS employs a local integral solution of the Boltzmann-BGK equation in calculating the numerical flux, while the DUGKS adopts a local discrete characteristic solution. By utilizing these multiscale techniques, it becomes possible to overcome the limitation of requiring a mesh size smaller than the mean free path of molecules. Furthermore, these multiscale approaches employ implicit discretization for the collision term, thereby addressing the limitation of needing a time step size smaller than the collision time.

In the scenario of UGKS, both the Boltzmann-BGK equation and the corresponding macroscopic governing equation are concurrently addressed. On the one hand, this simultaneous solution allows for predicting the equilibrium state at the new time level using solutions derived from the macroscopic governing equation. Gradoboev et al. [30] and Bishaev and Rykov [31] employed the macroscopic governing equation to speed up the calculation of some one-dimensional stationary problems. On the other hand, it also facilitates a straightforward implementation of fully implicit discretization in the multiscale approach. For example, Zhu et al. developed the implicit UGKS by employing the Lower-Upper Symmetric Gauss-Seidel (LU-SGS) method to solve both equations [32]. Similarly, Pan et al. constructed the implicit DUGKS [33]. Compared to their explicit counterparts, these implicit schemes exhibit significant acceleration in computation speed, particularly in near-continuum and continuum flow regimes.

It is evident in the multiscale approaches that the Boltzmann-BGK equation governs the solution in the rarefied flow regime, while the macroscopic governing equation takes precedence in the near-continuum and continuum flow regimes. Building upon this observation, an improved discrete velocity method (IDVM) was developed by integrating the local solution of the Boltzmann-BGK equation into the calculation of the macroscopic flux [34]. In the IDVM, the calculation of the numerical flux for the Boltzmann-BGK equation remains the same as in the conventional DVM to preserve its inherent simplicity. Additionally, by applying the LU-SGS method to address both the Boltzmann-BGK equation and the macroscopic governing equation, a fully implicit IDVM was developed and verified in all flow regimes.

To achieve faster convergence in the near-continuum and continuum flow regimes, the concept of inner iteration was recently introduced into the macroscopic governing equation. This approach allows for a more accurate prediction of the equilibrium state at the new time level, thereby accelerating the convergence rate. Several examples of this technique include the general synthetic iterative scheme (GSIS) [35], the multi-prediction implicit scheme [36], the IDVM with inner iteration [37], and the general implicit iterative method for UGKS [38]. Due to the significantly fewer number of conservative variables in the macroscopic governing equation compared to the discrete velocity distribution functions in the Boltzmann-BGK equation, the increase in computational cost for each outer loop iteration is minimal. Consequently, the overall computational cost can be substantially reduced by reducing the total number of iterations required for the outer loop.

Although several multiscale approaches have been developed to accelerate the convergence in DVM, a systematic comparative study in this field is still lacking. In this work, we aim to address this gap by focusing on the discrete velocity Boltzmann-BGK equation and undertaking a comparative analysis of three distinct multiscale schemes for its solution. The first scheme is a multiscale semi-implicit DVM (DVM-I). This approach utilizes the local discrete characteristic solution to calculate the numerical flux, addressing the limitation of mesh size being smaller than the mean free path of molecules. Additionally, it adopts a semi-implicit approach to discretize the collision term, thereby loosening the constraint of the time step size. The second scheme is a multiscale fully implicit DVM (DVM-II). Differing from DVM-I, DVM-II employs a fully implicit scheme to discretize the collision term and introduces the corresponding macroscopic governing equation to forecast the equilibrium state at the new time level. The third scheme is a multiscale fully implicit DVM with inner iteration (DVM-III). In this scheme, an inner iteration is incorporated to solve the macroscopic governing equation, aiming to enhance the accuracy of the predicted equilibrium state. To assess the performance of these multiscale schemes, we conduct an asymptotic analysis and numerical experiments for flows in the near-continuous/continuous flow regimes. By doing so, we aim to uncover the key factors that facilitate convergence acceleration in DVM calculations.

## 2. Discrete Velocity Boltzmann-BGK Equation

The original Boltzmann equation, an integral-differential equation with a complex collision term, poses significant challenges in practical engineering applications. In this study, our focus is on the Boltzmann equation integrated with the BGK model [3]. This amalgamation gives rise to the Boltzmann-BGK equation, which can be expressed as

(1) $$\frac{\partial f}{\partial t}+\xi \cdot \nabla f=\Omega =\frac{g-f}{\tau }$$

where f represents the distribution function defined in the physical space x, the molecular velocity space ξ, and the time t. Ω denotes the collision operator and τ is the collision time. The equilibrium state, denoted by g, is defined as:

(2) $$g=\frac{\rho }{{\left(2\pi R_{g}T\right)}^{3/2}}\exp\left[-\frac{c^{2}}{2R_{g}T}\right]$$

Here, ρ represents the density, T signifies the temperature, c=ξ−u stands for the molecular thermal velocity vector, u denotes the mean flow velocity vector, c=|c| symbolizes the magnitude of c, and $R_{g}$ represents the gas constant. To solve Equation (1) using the DVM, it is necessary to truncate and discretize the molecular velocity space into a series of discrete velocity points. This discretization process results in

(3) $$\frac{\partial f_{\alpha }}{\partial t}+\xi_{\alpha }\cdot \nabla f_{\alpha }=\Omega_{\alpha }=\frac{g_{\alpha }-f_{\alpha }}{\tau },\alpha =1,\cdots ,N_{V}$$

where, $N_{V}$ represents the total number of discrete velocity points and the subscript α denotes the index in the discrete velocity space.

With Equation (1), we can derive the corresponding macroscopic governing equation of conservation laws through moment integration. By multiplying Equation (1) by the microscopic conservative moment $\Psi ={\left(1,\xi ,\xi^{2}/2\right)}^{T}$ and subsequently integrating the resulting equation in the molecular velocity space, we can obtain:

(4) $$\frac{\partial W}{\partial t}+\nabla \cdot F=0$$

(5) $$W={\left(\rho ,\rho u,\rho E\right)}^{T}={\langle \Psi f\rangle }_{\alpha }={\langle \Psi g\rangle }_{\alpha }$$

(6) $$F={\left(F_{\rho },F_{\rho u},F_{\rho E}\right)}^{T}={\langle \xi \Psi f\rangle }_{\alpha }$$

where, W and F represent the conservative flow variables and fluxes, respectively. The notation ${\langle f\rangle }_{\alpha }=\sum_{N_{V}}f_{\alpha }$ defines the numerical quadrature of distribution functions across the entire discrete molecular velocity space. As reported in previous studies [8,39], the conservation with respect to the collision integral plays a crucial role in ensuring the stability and accuracy of the DVM. However, the primary focus of this paper is to investigate the impact of different discretization strategies for the collision term on the computational efficiency of the DVM. To address the potential adverse effects caused by numerical quadrature errors, we will employ a relatively large number of points in the discretization of the molecular velocity space during the numerical tests.

## 3. Three Versions of DVM

To investigate the key factors that contribute to convergence acceleration in DVM calculations, this section introduces and compares three versions of DVM. In these schemes, similar to the DUGKS [27,28,29], the local discrete characteristic solution of the Boltzmann-BGK equation is employed to calculate the numerical flux, addressing the limitation associated with mesh size. This local solution is obtained by integrating Equation (1) from $t^{n}=0$ to $t^{n}+\Delta t_{p}/2$ along the characteristic line and approximating the collision term using the trapezoidal rule. By doing so, we can obtain

(7) $$f_{\alpha }\left(x_{ij},h\right)=\frac{2\tau -h}{2\tau +h}f_{\alpha }\left(x_{ij}-\xi_{\alpha }h,0\right)+\frac{h}{2\tau +h}\left(g_{\alpha }\left(x_{ij}-\xi_{\alpha }h,0\right)+g_{\alpha }\left(x_{ij},h\right)\right)$$

where $x_{ij}$ denotes the mid-point of the cell interface. $h=\Delta t_{p}/2$ signifies the half-time step size and $\Delta t_{p}$ stands for the physical time step used only for flux reconstruction to avoid extrapolation, which is defined by

(8) $$\Delta t_{p}=\sigma_{p}\frac{V}{\xi_{x,\max}\Delta S_{x}+\xi_{y,\max}\Delta S_{y}}$$

Here, $\xi_{x,\max}$ and $\xi_{y,\max}$ represent the maximum molecular velocities in the *x*- and *y*-directions, respectively. V signifies the volume of the control cell. $\Delta S_{x}$ and $\Delta S_{y}$ are the projected areas of the control volume in the *y*- and *x*-directions, respectively. $\sigma_{p}$ is the Courant–Friedrichs–Lewy (CFL) number, which is set as $\sigma_{p}=0.95$.

To fully determine the distribution function at the cell interface $f_{\alpha }\left(x_{ij},h\right)$, it is essential to pre-calculate the discrete distribution function at the surrounding point of the cell interface $f_{a}\left(x_{ij}-\xi_{\alpha }h,0\right)$, the equilibrium state at the surrounding point of the cell interface $g_{\alpha }\left(x_{ij}-\xi_{\alpha }h,0\right)$, and the equilibrium state at the mid-point of the cell interface $g_{\alpha }\left(x_{ij},h\right)$. For the calculation of $f_{a}\left(x_{ij}-\xi_{\alpha }h,0\right)$ and $g_{\alpha }\left(x_{ij}-\xi_{\alpha }h,0\right)$, a second-order interpolation scheme employing van Leer’s slope limiter is utilized. Taking ϕ to represent either $f_{a}$ or $g_{\alpha }$, we can obtain:

(9) $$\phi \left(x_{ij}-\xi_{\alpha }h,0\right)=\{\begin{array}{c} \phi^{L}\left(x_{ij},0\right)-h\xi_{\alpha }\cdot \nabla \phi \left(x_{i},0\right)L\left(\phi ,x_{i}\right),\;n_{ij}\cdot \xi \geq 0\\ \phi^{R}\left(x_{ij},0\right)-h\xi_{\alpha }\cdot \nabla \phi \left(x_{j},0\right)L\left(\phi ,x_{j}\right),\;n_{ij}\cdot \xi <0 \end{array}$$

where $\phi^{L}$ and $\phi^{R}$ represent the interfacial states of ϕ at the left and right sides, respectively. $\nabla \phi \left(x_{i},0\right)$ and $\nabla \phi \left(x_{j},0\right)$ denote the derivatives of ϕ at the left and right cells. $L\left(\phi ,x_{i}\right)$ and $L\left(\phi ,x_{j}\right)$ are the corresponding slope limiter functions.

For the calculation of $g_{\alpha }\left(x_{ij},h\right)$, the macroscopic flow variables at the cell interface are required. This can be achieved by computing the conservative moments on both sides of Equation (7), which yields:

(10) $$W\left(x_{ij},h\right)=\frac{2\tau -h}{2\tau +h}{\langle \Psi f\left(x_{ij}-\xi_{\alpha }h,0\right)\rangle }_{a}+\frac{h}{2\tau +h}\left\{{\langle \Psi g\left(x_{ij}-\xi_{\alpha }h,0\right)\rangle }_{a}+W\left(x_{ij},h\right)\right\}$$

The above equation can be rearranged as follows

(11) $$W\left(x_{ij},h\right)=\frac{2\tau -h}{2\tau }{\langle \Psi f\left(x_{ij}-\xi_{\alpha }h,0\right)\rangle }_{a}+\frac{h}{2\tau }{\langle \Psi g\left(x_{ij}-\xi_{\alpha }h,0\right)\rangle }_{a}$$

Since $f_{a}\left(x_{ij}-\xi_{\alpha }h,0\right)$ and $g_{a}\left(x_{ij}-\xi_{\alpha }h,0\right)$ have been obtained previously, $W\left(x_{ij},h\right)$ as well as $g_{\alpha }\left(x_{ij},h\right)$ can be computed explicitly.

Once the local discrete characteristic solution of the Boltzmann-BGK equation at the cell interface is determined, calculating the numerical fluxes for both the Boltzmann-BGK equation and the corresponding macroscopic governing equation becomes straightforward. This facilitates the evolution of both the discrete distribution functions and the conservative variables. In the following subsection, three different strategies are employed to advance the evolution of these quantities.

### 3.1. Semi-Implicit DVM (DVM-I)

The first multiscale approach is the semi-implicit DVM, similar to the work of Yang and Huang [18], where the equilibrium state in the collision term is calculated using the flow variables at the current time level, while other parts of the Boltzmann-BGK equation are discretized implicitly. This leads to the following discretized equation:

(12) $$\frac{f_{\alpha }^{n+1}-f_{\alpha }^{n}}{\Delta t^{n}}+\xi_{\alpha }\cdot \nabla f_{\alpha }^{n+1}=\frac{g_{\alpha }^{n}-f_{\alpha }^{n+1}}{\tau^{n}}$$

where Δt represents the time step size for the evolution of the distribution function, determined by the CFL condition. But different from Equation (8), the CFL number for the calculation of Δt can be chosen larger than one to attain faster convergence. Integrating Equation (12) over a control volume $V_{i}$, we can obtain a finite volume discretization of the Boltzmann-BGK equation as follows:

(13) $$\frac{V_{i}}{\Delta t_{i}^{n}}\left(f_{i,\alpha }^{n+1}-f_{i,\alpha }^{n}\right)+\sum_{j\in N\left(i\right)}n_{ij}\cdot \xi_{\alpha }S_{ij}f_{ij,\alpha }^{n+1}=\frac{V_{i}}{\tau_{i}^{n}}\left(g_{i,\alpha }^{n}-f_{i,\alpha }^{n+1}\right)$$

where N(i) is the set of neighboring cells of the cell i, $S_{ij}$ stands for the area of the interface shared by the cells i and j, and $n_{ij}$ signifies the unit normal vector of the shared interface pointing from cell i to cell j.

By introducing the incremental $\Delta f_{i,\alpha }^{n}=f_{i,\alpha }^{n+1}-f_{i,\alpha }^{n}$ into Equation (13), we can obtain:

(14) $$\left(\frac{V_{i}}{\Delta t_{i}^{n}}+\frac{V_{i}}{\tau_{i}^{n}}\right)\Delta f_{i,\alpha }^{n}+\sum_{j\in N\left(i\right)}n_{ij}\cdot \xi_{\alpha }S_{ij}\Delta f_{ij,\alpha }^{n}={\mathrm{RHS}}_{i,\alpha }^{n}$$

with the right-hand side of:

(15) $${\mathrm{RHS}}_{i,\alpha }^{n}=\frac{V_{i}}{\tau_{i}^{n}}\left(g_{i,\alpha }^{n}-f_{i,\alpha }^{n}\right)-\sum_{j\in N\left(i\right)}n_{ij}\cdot \xi_{\alpha }S_{ij}f_{ij,\alpha }^{n}$$

Since the distribution function at the cell interface has been determined by Equation (7), the right-hand side of Equation (14) can be calculated explicitly. For the reconstruction of $\Delta f_{ij,\alpha }^{n}$, the first-order upwind scheme is employed, i.e.,

(16) $$\Delta f_{ij,\alpha }^{n}=\frac{1}{2}\left(\Delta f_{i,\alpha }^{n}+\Delta f_{j,\alpha }^{n}\right)+\frac{1}{2}sign\left(n_{ij}\cdot \xi_{\alpha }\right)\left(\Delta f_{i,\alpha }^{n}-\Delta f_{j,\alpha }^{n}\right)$$

where $sign\left(n_{ij}\cdot \xi_{\alpha }\right)$ is the sign function, $sign\left(n_{ij}\cdot \xi_{\alpha }\right)=1$ for $n_{ij}\cdot \xi_{\alpha }\geq 0$ and $sign\left(n_{ij}\cdot \xi_{\alpha }\right)=-1$ for $n_{ij}\cdot \xi_{\alpha }<0$. Substituting Equation (16) into Equation (14) and applying the LU-SGS method to solve the resultant equation [40], the incremental $\Delta f_{i,\alpha }^{n}$ can be obtained by the following forward and backward sweepings:

(17) $$D_{i,\alpha }^{n}{\bar{\Delta f}}_{i,\alpha }^{n}+\frac{1}{2}\sum_{j\in L\left(i\right)}\left(n_{ij}\cdot \xi_{\alpha }-\left|n_{ij}\cdot \xi_{\alpha }\right|\right)S_{ij}{\bar{\Delta f}}_{j,a}^{n}={\mathrm{RHS}}_{i,\alpha }^{n}$$

(18) $$D_{i,\alpha }^{n}\Delta f_{i,\alpha }^{n}+\frac{1}{2}\sum_{j\in U\left(i\right)}\left(n_{ij}\cdot \xi_{\alpha }-\left|n_{ij}\cdot \xi_{\alpha }\right|\right)S_{ij}\Delta f_{j,a}^{n}=D_{i,\alpha }^{n}{\bar{\Delta f}}_{i,\alpha }^{n}$$

with:

$$D_{i,\alpha }^{n}=\frac{V_{i}}{\Delta t_{i}^{n}}+\frac{V_{i}}{\tau_{i}^{n}}+\frac{1}{2}\sum_{j\in N\left(i\right)}\left|n_{ij}\cdot \xi_{\alpha }\right|S_{ij}$$

where L(i) is the subset of N(i) with elements less than i, while U(i) is the subset of N(i) with elements larger than i. ${\bar{\Delta f}}_{i,\alpha }^{n}$ denotes the intermediate result of the forward sweep. Upon obtaining the incremental $\Delta f_{i,\alpha }^{n}$, the discretized distribution function can be updated by $f_{i,\alpha }^{n+1}=f_{i,\alpha }^{n}+\Delta f_{i,\alpha }^{n}$ and the macroscopic flow variables can be obtained by Equations (5) and (6).

### 3.2. Fully Implicit DVM (DVM-II)

Unlike the semi-implicit DVM, the fully implicit DVM calculates the equilibrium state within the collision term using the predicted solution from the corresponding macroscopic governing equation at the new time level. This strategy has found widespread use in the development of the fully implicit scheme, culminating in the following discretized Boltzmann-BGK equation [32,33,34]

(19) $$\frac{f_{\alpha }^{n+1}-f_{\alpha }^{n}}{\Delta t^{n}}+\xi_{\alpha }\cdot \nabla f_{\alpha }^{n+1}=\frac{{\hat{g}}_{\alpha }^{n+1}-f_{\alpha }^{n+1}}{\tau^{n}}$$

Following the solution process of Equation (12), Equation (19) can be expressed as:

(20) $$\left(\frac{V_{i}}{\Delta t_{i}^{n}}+\frac{V_{i}}{\tau_{i}^{n}}+\frac{1}{2}\sum_{j\in N\left(i\right)}\left|n_{ij}\cdot \xi_{\alpha }\right|S_{ij}\right)\Delta f_{i,\alpha }^{n}+\frac{1}{2}\sum_{j\in N\left(i\right)}\left(n_{ij}\cdot \xi_{\alpha }-\left|n_{ij}\cdot \xi_{\alpha }\right|\right)S_{ij}\Delta f_{j,a}^{n}={\mathrm{RHS}}_{i,\alpha }^{n}$$

with the right-hand side given by:

(21) $${\mathrm{RHS}}_{i,\alpha }^{n}=\frac{V_{i}}{\tau_{i}^{n}}\left({\hat{g}}_{i,\alpha }^{n+1}-f_{i,\alpha }^{n}\right)-\sum_{j\in N\left(i\right)}n_{ij}\cdot \xi_{\alpha }S_{ij}f_{ij,\alpha }^{n}$$

Equation (20) can be easily solved using the LU-SGS method. However, before we proceed to solve this equation, it is necessary to determine the predicted equilibrium state ${\hat{g}}_{i,\alpha }^{n+1}$.

As we know, the macroscopic governing equation can be derived from the Boltzmann-BGK equation through moment integration. By multiplying Equation (19) by the microscopic conservative moment $\Psi ={\left(1,\xi ,\xi^{2}/2\right)}^{T}$ and subsequently integrating the resulting equation across the molecular velocity space, we can derive:

(22) $$\frac{{\hat{W}}^{n+1}-W^{n}}{\Delta t^{n}}+\nabla \cdot {\hat{F}}^{n+1}=0$$

By introducing the incremental $\Delta {\hat{W}}^{n}={\hat{W}}^{n+1}-W^{n}$ and integrating Equation (22) over a control volume $V_{i}$, we can obtain:

(23) $$\frac{V_{i}}{\Delta t_{i}^{n}}\Delta {\hat{W}}_{i}^{n}=-{\hat{R}}_{i}^{n+1}=-\sum_{j\in N\left(i\right)}n_{ij}\cdot {\hat{F}}_{ij}^{n+1}S_{ij}$$

To solve the aforementioned equation, it is necessary to linearize the residual term ${\hat{R}}_{i}^{n+1}$ into the following form:

(24) $${\hat{R}}_{i}^{n+1}=R_{i}^{n}+\Delta {\hat{R}}_{i}^{n}=\sum_{j\in N\left(i\right)}{\langle n_{ij}\cdot \xi \Psi f_{ij}^{n}\rangle }_{\alpha }S_{ij}+\sum_{j\in N\left(i\right)}\frac{\partial R_{i}^{n}}{\partial W_{j}^{n}}\Delta W_{j}^{n}$$

Since the distribution function at the cell interface has been determined by Equation (7), the first term on the right-hand side of Equation (24) can be calculated explicitly.

For the calculation of the last term of Equation (24), the Euler equations-based flux splitting method is adopted:

(25) $$\sum_{j\in N\left(i\right)}\frac{\partial R_{i}^{n}}{\partial W_{j}^{n}}\Delta W_{j}^{n}=\frac{1}{2}\sum_{j\in N\left(i\right)}\left[n_{ij}\cdot \left(\Delta F_{c,i}^{n}+\Delta F_{c,j}^{n}\right)+r_{ij}^{n}\left(\Delta W_{i}^{n}-\Delta W_{j}^{n}\right)\right]S_{ij}$$

where,

$$\Delta F_{c,j}^{n}=F_{c}\left(W_{j}^{n}+\Delta W_{j}^{n}\right)-F_{c}\left(W_{j}^{n}\right)$$

$$r_{ij}^{n}=\left(\left|n_{ij}\cdot u_{ij}^{n}\right|+c_{s,ij}^{n}\right)+\max\left(\frac{4}{3\rho_{ij}^{n}},\frac{\gamma }{\rho_{ij}^{n}}\right)\frac{\mu_{ij}^{n}}{Pr\left|x_{j}-x_{i}\right|}$$

Here, $F_{c}={\left(\rho u,\rho uu+pI,\left(\rho E+p\right)u\right)}^{T}$ denotes the convective flux of the macroscopic governing equation, $c_{s}$ represents the sound speed, γ signifies the specific heat ratio, Pr stands for the Prandtl number, and $x_{i}$ and $x_{j}$ represent the centroids of cell *i* and cell *j*, respectively. By substituting Equations (24) and (25) into Equation (23) and applying the LU-SGS method to solve the resultant equation [40], we can obtain:

(26) $$D_{i}^{n}{\bar{\Delta W}}_{i}^{n}+\frac{1}{2}\sum_{j\in L\left(i\right)}\left[n_{ij}\cdot \Delta F_{c,j}^{n}-r_{ij}^{n}{\bar{\Delta W}}_{j}^{n}\right]S_{ij}=-R_{i}^{n}$$

(27) $$D_{i}^{n}\Delta {\hat{W}}_{i}^{n}+\frac{1}{2}\sum_{j\in U\left(i\right)}\left[n_{ij}\cdot \Delta F_{c,j}^{n}-r_{ij}^{n}\Delta {\hat{W}}_{j}^{n}\right]S_{ij}=D_{i}^{n}{\bar{\Delta W}}_{i}^{n}$$

where $D_{i}^{n}=\frac{V_{i}}{\Delta t_{i}^{n}}+\frac{1}{2}\sum_{j\in N\left(i\right)}r_{ij}^{n}S_{ij}$ and ${\bar{\Delta W}}_{i}^{n}$ represent the intermediate result of the forward sweep. Once the incremental $\Delta {\hat{W}}_{i}^{n}$ is obtained, the predicted conservative variables can be calculated by ${\hat{W}}^{n+1}=W^{n}+\Delta {\hat{W}}^{n}$ and the predicted equilibrium state can be computed by substituting ${\hat{W}}^{n+1}$ into Equation (2).

### 3.3. Fully Implicit DVM with Inner Iteration (DVM-III)

In DVM-II, it is observed that both the Boltzmann-BGK equation and the corresponding macroscopic governing equation iterate only once at each time step. In the near-continuum and continuum flow regimes, the macroscopic governing equation tends to approach the Navier–Stokes equation. However, the flux Jacobian of the macroscopic governing equation is approximated using the Euler equations-based flux splitting method, which might lead to inaccuracies in predicting the equilibrium state. To enhance the accuracy of the predicted equilibrium state in the near-continuum and continuum flow regimes, similar to prior studies [35,36,37,38], an inner iteration is introduced to solve the macroscopic governing equation. To achieve this goal, a time derivative term with respect to the pseudo-time variable η is added on the left-hand side of Equation (23) as follows:

(28) $$V_{i}\frac{\partial {\hat{W}}_{i}^{n+1}}{\partial \eta }+\frac{V_{i}}{\Delta t_{i}^{n}}\left({\hat{W}}_{i}^{n+1}-W_{i}^{n}\right)=-\left(R_{i}^{n}+\Delta {\hat{R}}_{i}^{n}\right)$$

In DVM-III, the flux Jacobian of the macroscopic governing equation $\Delta {\hat{R}}_{i}^{n}$ is calculated by taking the difference between the fluxes of the Navier–Stokes equation at the current time level and the new time level. This can be expressed as:

(29) $$\Delta {\hat{R}}_{i}^{n}=\sum_{j\in N\left(i\right)}n_{ij}\cdot \left({\hat{F}}_{ij}^{n+1}-F_{ij}^{n}\right)S_{ij}\approx \sum_{j\in N\left(i\right)}n_{ij}\cdot \left({\hat{F}}_{ij,NS}^{n+1}-F_{ij,NS}^{n}\right)S_{ij}$$

where $F_{ij,NS}^{n}$ and ${\hat{F}}_{ij,NS}^{n+1}$ represent the numerical fluxes of the Navier–Stokes equation at the current time level and the new time level, respectively. This equation signifies that the accurate difference in the numerical fluxes of the macroscopic governing equation, computed using the distribution function, is estimated by the disparity in numerical fluxes of the Navier–Stokes equation. This approximation is considered more reasonable than the Euler equations-based flux splitting method, particularly in the context of near-continuum and continuum flow regimes [36,37,38].

By discretizing the time derivative of Equation (28) with the backward Euler scheme and substituting Equation (29) into the resultant equation, we can obtain:

(30) $$\left(\frac{V_{i}}{\Delta \eta_{i}^{n+1,m}}+\frac{V_{i}}{\Delta t_{i}^{n}}\right)\Delta {\hat{W}}_{i}^{n+1,m}+\sum_{j\in N\left(i\right)}n_{ij}\cdot \Delta {\hat{F}}_{ij,NS}^{n+1,m}S_{ij}=-{\hat{\mathbb{R}}}_{i}^{n+1,m}$$

with:

(31) $${\hat{\mathbb{R}}}_{i}^{n+1,m}=R_{i}^{n}+\sum_{j\in N\left(i\right)}n_{ij}\cdot \left({\hat{F}}_{ij,NS}^{n+1,m}-F_{ij,NS}^{n}\right)S_{ij}+\frac{V_{i}}{\Delta t_{i}^{n}}\left({\hat{W}}_{i}^{n+1,m}-W_{i}^{n}\right)$$

Here, $\Delta {\hat{W}}_{i}^{n+1,m}={\hat{W}}_{i}^{n+1,m+1}-{\hat{W}}_{i}^{n+1,m}$ and $\Delta {\hat{F}}_{ij,NS}^{n+1,m}={\hat{F}}_{ij,NS}^{n+1,m+1}-{\hat{F}}_{ij,NS}^{n+1,m}$ represent the increments in the predicted conservative variable vector and the flux vector of the Navier–Stokes equation at the pseudo-time level m of the inner iteration.

During the inner iteration process, as the solution converges, the values of $\Delta {\hat{W}}_{i}^{n+1,m}$ and $\Delta {\hat{F}}_{ij,NS}^{n+1,m}$ tend to approach zero. Thus, the Euler equations-based flux splitting method can be employed to calculate the flux Jacobian in the inner iteration,

(32) $$\sum_{j\in N\left(i\right)}n_{ij}\cdot \Delta {\hat{F}}_{ij,NS}^{n+1,m}S_{ij}=\frac{1}{2}\sum_{j\in N\left(i\right)}\left[n_{ij}\cdot \left(\Delta {\hat{F}}_{c,i}^{n+1,m}+\Delta {\hat{F}}_{c,j}^{n+1,m}\right)+r_{ij}^{n+1,m}\left(\Delta {\hat{W}}_{i}^{n+1,m}-\Delta {\hat{W}}_{j}^{n+1,m}\right)\right]S_{ij}$$

where $\Delta {\hat{F}}_{c,j}^{n+1,m}=F_{c}\left({\hat{W}}_{j}^{n+1,m}+\Delta {\hat{W}}_{j}^{n+1,m}\right)-F_{c}\left({\hat{W}}_{j}^{n+1,m}\right)$. Substituting Equation (32) into Equation (30), we can derive:

(33) $$G_{i}^{n+1,m}\Delta {\hat{W}}_{i}^{n+1,m}+\frac{1}{2}\sum_{j\in N\left(i\right)}\left[n_{ij}\cdot \Delta {\hat{F}}_{c,j}^{n+1,m}-r_{ij}^{n+1,m}\Delta {\hat{W}}_{j}^{n+1,m}\right]S_{ij}=-{\hat{\mathbb{R}}}_{i}^{n+1,m}$$

with:

$$G_{i}^{n+1,m}=\frac{V_{i}}{\Delta \eta_{i}^{n+1,m}}+\frac{V_{i}}{\Delta t_{i}^{n}}+\frac{1}{2}\sum_{j\in N\left(i\right)}r_{ij}^{n+1,m}S_{ij}$$

Equation (33) can be easily resolved through the LU-SGS method [40]. At the beginning of the inner iteration, the initial values of the conservative variables and fluxes are set as ${\hat{W}}_{i}^{n+1,m=1}={\hat{W}}_{i}^{n}$ and ${\hat{F}}_{ij,NS}^{n+1,m=1}=F_{ij,NS}^{n}$. At the conclusion of the inner iteration, we can set ${\hat{W}}_{i}^{n+1}={\hat{W}}_{i}^{n,m=M}$, where M signifies the iteration number of the inner iteration and it is chosen as 50 in this work. In addition, for simplicity, the time step size in the pseudo-time level is chosen as $\Delta \eta_{i}^{n+1,m}=\Delta t_{i}^{n}$.

### 3.4. Comparison of Three Schemes

The three schemes mentioned above all entail calculating the numerical flux using the local discrete characteristic solution of the Boltzmann-BGK equation. Additionally, they employ either a semi-implicit or fully implicit scheme to discretize the collision term. This allows for a mesh size unrestricted by the mean free path of molecules and a time step size not limited by the collision time. The primary distinction among these schemes lies in how they incorporate the solution of the macroscopic governing equation. In DVM-II, the solution of the macroscopic governing equation is introduced, while in DVM-III, an inner iteration is used to solve the macroscopic governing equation. However, considering that the computational cost of solving the macroscopic governing equation is significantly lower than that of the Boltzmann-BGK equation, the increased computational cost of DVM-II and DVM-III in each time step for evolving the discrete distribution function is negligible. In this subsection, we will qualitatively compare the three schemes in terms of the collisionless limit and continuum limit. For convenient comparison, let us rewrite the evolution equations of the three schemes as follows:

In DVM-I, only the evolution equation of the distribution function is involved. This equation can be written as:

(34) $$f_{i,\alpha }^{n+1}=\frac{\Delta t_{i}^{n}}{\tau_{i}^{n}+\Delta t_{i}^{n}}g_{i,\alpha }^{n}+\frac{\tau_{i}^{n}}{\tau_{i}^{n}+\Delta t_{i}^{n}}f_{i,\alpha }^{n}-\frac{\tau_{i}^{n}\Delta t_{i}^{n}}{\tau_{i}^{n}+\Delta t_{i}^{n}}\frac{1}{V_{i}}\sum_{j\in N\left(i\right)}n_{ij}\cdot \xi_{\alpha }S_{ij}f_{ij,\alpha }^{n+1}$$

In DVM-II, both the evolution equations of the distribution function and the macroscopic conservative variables are involved. These equations can be expressed as:

(35) $$f_{i,\alpha }^{n+1}=\frac{\Delta t_{i}^{n}}{\tau_{i}^{n}+\Delta t_{i}^{n}}{\hat{g}}_{\alpha }^{n+1}+\frac{\tau_{i}^{n}}{\tau_{i}^{n}+\Delta t_{i}^{n}}f_{i,\alpha }^{n}-\frac{\tau_{i}^{n}\Delta t_{i}^{n}}{\tau_{i}^{n}+\Delta t_{i}^{n}}\frac{1}{V_{i}}\sum_{j\in N\left(i\right)}n_{ij}\cdot \xi_{\alpha }S_{ij}f_{ij,\alpha }^{n+1}$$

(36) $${\hat{W}}^{n+1}=W^{n}-\frac{\Delta t^{n}}{V_{i}}\left(\sum_{j\in N\left(i\right)}{\langle n_{ij}\cdot \xi \Psi f_{ij}^{n}\rangle }_{\alpha }S_{ij}+\sum_{j\in N\left(i\right)}\frac{\partial R_{i}^{n}}{\partial W_{j}^{n}}\Delta W_{j}^{n}\right)$$

(37) $$\sum_{j\in N\left(i\right)}\frac{\partial R_{i}^{n}}{\partial W_{j}^{n}}\Delta W_{j}^{n}=\frac{1}{2}\sum_{j\in N\left(i\right)}\left[n_{ij}\cdot \left(\Delta F_{c,i}^{n}+\Delta F_{c,j}^{n}\right)+r_{ij}^{n}\left(\Delta W_{i}^{n}-\Delta W_{j}^{n}\right)\right]S_{ij}$$

In DVM-III, the evolution equations of the distribution function and the macroscopic conservative variables maintain the same forms as Equations (35) and (36), with the distinction that the flux Jacobian is calculated by the disparity of fluxes of Navier–Stokes equation:

(38) $$\sum_{j\in N\left(i\right)}\frac{\partial R_{i}^{n}}{\partial W_{j}^{n}}\Delta W_{j}^{n}=\sum_{j\in N\left(i\right)}n_{ij}\cdot \left({\hat{F}}_{ij,NS}^{n+1}-F_{ij,NS}^{n}\right)S_{ij}$$

Note that, in DVM-II and DVM-III, the predicted equilibrium state ${\hat{g}}_{\alpha }^{n+1}$ is calculated from the solution of the macroscopic governing equation ${\hat{W}}^{n+1}$.

(1) **Collisionless limit**

In the collisionless limit, where the collision time is significantly larger than the time step size, τ≫Δt, the evolution equation of the discrete distribution function for all three schemes can be simplified as follows:

(39) $$f_{i,\alpha }^{n+1}=f_{i,\alpha }^{n}-\frac{\Delta t_{i}^{n}}{V_{i}}\sum_{j\in N\left(i\right)}n_{ij}\cdot \xi_{\alpha }S_{ij}f_{ij,\alpha }^{n+1}$$

This simplification disregards any equilibrium state and can be considered as a solution to the following collisionless Boltzmann equation.

(40) $$\frac{\partial f_{\alpha }}{\partial t}+\xi_{\alpha }\cdot \nabla f_{\alpha }=0$$

For DVM-II and DVM-III, even though the evolution of macroscopic conservative variables is involved, the predicted equilibrium state does not directly impact the evolution of the discrete distribution function. Hence, all three schemes can provide reasonable results in the collisionless limit.

(2) **Continuum limit**

In the continuum limit, where the collision time is significantly less than the time step size, τ≪Δt, the evolution equations of the discrete distribution function for DVM-I, DVM-II, and DVM-III can be respectively simplified as follows:

For DVM-I:

(41) $$f_{i,\alpha }^{n+1}=g_{i,\alpha }^{n}-\frac{\tau_{i}^{n}}{V_{i}}\sum_{j\in N\left(i\right)}n_{ij}\cdot \xi_{\alpha }S_{ij}f_{ij,\alpha }^{n+1}$$

For DVM-II and -III:

(42) $$f_{i,\alpha }^{n+1}={\hat{g}}_{\alpha }^{n+1}-\frac{\tau_{i}^{n}}{V_{i}}\sum_{j\in N\left(i\right)}n_{ij}\cdot \xi_{\alpha }S_{ij}f_{ij,\alpha }^{n+1}$$

It can be observed that the effective time step size for the evolution of the discrete distribution function is the collision time. However, the initial values (the predicted equilibrium state) differ among the three schemes. A more accurate initial value leads to a faster convergence rate.

In order to achieve a more precise predicted equilibrium state, the evolution equation of the predicted macroscopic conservative variables (as shown in Equation (36)) is introduced in DVM-II and DVM-III. It is evident that the effective time step size of Equation (36) is much larger than the collision time in this scenario. This implies that the macroscopic governing equation takes precedence in the continuum limit. Consequently, the flow field can be accurately described by the Navier–Stokes equation in this regime. Naturally, DVM-III yields a more accurate predicted equilibrium state compared to DVM-II since the flux Jacobian is calculated using the Navier–Stokes equation in this method.

## 4. Numerical Examples

In this section, we perform a numerical investigation to evaluate the performance of the three schemes and uncover the key to accelerating convergence in DVM calculations. The evaluation encompasses six diverse test examples with varying Knudsen/Reynolds numbers, effectively covering a broad spectrum of flow regimes. These examples include Couette flow, heat transfer between parallel plates, force-driven Poiseuille flow, lid-driven cavity flow, flow around a NACA0012 airfoil, and flow in a planar microchannel. All computations are carried out on a PC equipped with an Intel(R) Xeon(R) Gold 6226R CPU operating at 2.9 GHz. No parallelization techniques are employed during these computations.

### 4.1. Case 1: Couette Flow

The first test case involves the Couette flow, consisting of two vertically positioned plates situated at $x_{L}=0$ and $x_{R}=1$. Both plates have the same temperature of $T_{0}=1$ and different constant vertical velocities: $v_{L}=-0.25$ for the left plate and $v_{R}=0.25$ for the right plate. The Maxwellian diffuse boundary condition is employed for the left and right boundaries, while the periodic boundary condition is adopted for the upper and lower boundaries. The Knudsen number for this case is defined as:

(43) $$Kn=\frac{\sqrt{\pi }\mu_{0}}{\rho_{0}{\left(2R_{g}T_{0}\right)}^{1/2}L_{0}}$$

where $\rho_{0}=1$ is the reference density, $L_{0}=x_{R}-x_{L}$ denotes the reference length, $\mu_{0}$ represents the reference viscosity, and $R_{g}=0.5$ signifies the specific gas constant. The dynamic viscosity is determined using the following intermolecular interaction model:

(44) $$\mu =\mu_{0}{\left(\frac{T}{T_{0}}\right)}^{w}$$

with the viscosity index w=0.81. In the simulation, we consider five different Knudsen numbers: *Kn* = 0.001, 0.01, 0.1, 1, and 10. The physical space is discretized non-uniformly into 100 cells in the *x*-direction and 5 cells in the *y*-direction. For the discretization of the molecular velocity space, we use the Gauss–Hermite quadrature with 28 × 28 mesh points for the cases of *Kn* = 0.001, 0.01, and 0.1. In the cases of *Kn* = 1 and 10, we utilize the Newton–Cotes quadrature with 101 × 101 mesh points uniformly distributed in the range of [−6, 6] × [−6, 6].

**Figure 1:**
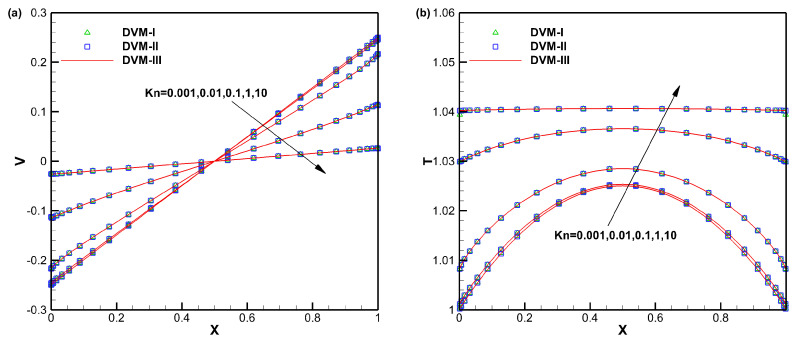
Velocity (**a**) and temperature (**b**) distributions along the *x*-direction for Couette flow with different Knudsen numbers.

**Figure 2:**
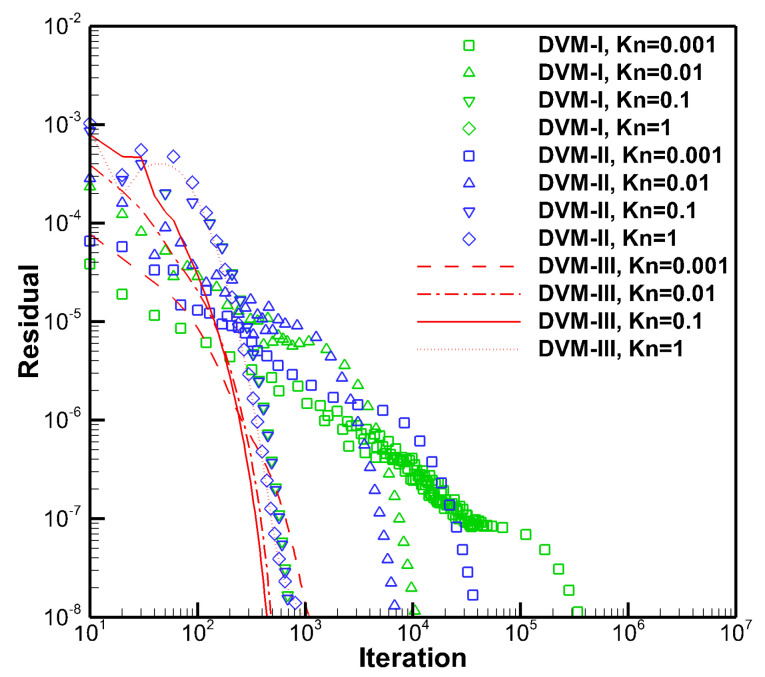
Convergence history of DVM-I, DVM-II, and DVM-III for Couette flow with different Knudsen numbers.

The converged velocity and temperature distributions along the *x*-direction are shown in Figure 1, demonstrating good agreement among DVM-I, DVM-II, and DVM-III. Figure 2 compares the convergence history of the three schemes. It is evident that, in the rarefied flow regime, all three schemes converge similarly. However, in the near-continuum and continuum flow regimes, the DVM-III achieves the fastest convergence, followed by the DVM-II, while the DVM-I exhibits slower convergence. This indicates that the fully implicit discretization of the collision term is pivotal for accelerating convergence in DVM calculations, and a more accurate prediction of the equilibrium state leads to a faster convergence rate. The quantitative comparison of the computational cost of the three schemes is tabulated in Table 1. Since the computational cost of solving the macroscopic governing equation is significantly lower than that of the Boltzmann-BGK equation, DVM-II achieves approximately one order of magnitude acceleration compared to DVM-I. Additionally, DVM-III achieves an additional order of magnitude acceleration compared to DVM-II.

### 4.2. Case 2: Heat Transfer between Two Parallel Plates

The second test case involves heat transfer between two stationary parallel plates. The geometry configuration and mesh in the physical space for this test case are the same as those in the first test case. The left plate is maintained at a fixed temperature of *T_L_* = 0.75, while the right plate is kept at a fixed temperature of *T_R_* = 1.25. The Knudsen number is determined by Equation (43) with the reference density of $\rho_{0}=1$ and reference temperature of $T_{0}=1$, and the dynamic viscosity is calculated using Equation (44) with the viscosity index w=0.81. In the simulation, we consider five different Knudsen numbers: *Kn* = 0.001, 0.01, 0.1, 1, and 10, which cover all the flow regimes. For each Knudsen number, the molecular velocity space discretization follows the same strategy as that in the first test case.

**Figure 3:**
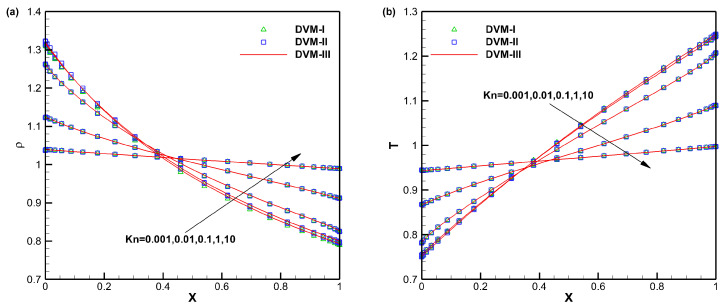
Density (**a**) and temperature (**b**) distributions along the *x*-direction for heat transfer between two parallel plates with different Knudsen numbers.

**Figure 4:**
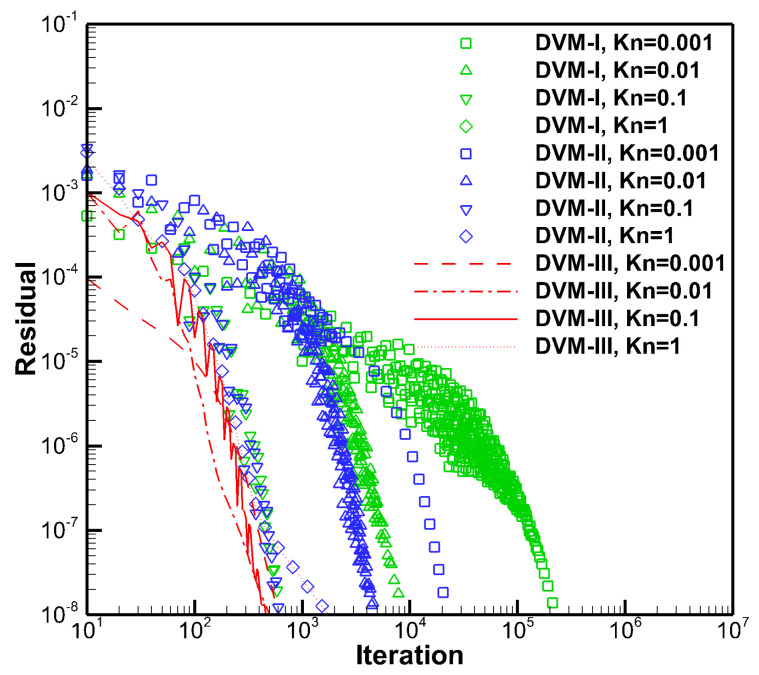
Convergence history of DVM-I, DVM-II, and DVM-III for heat transfer between two parallel plates with different Knudsen numbers.

Figure 3 illustrates a comparison of the converged density and temperature distributions along the *x*-direction obtained using DVM-I, DVM-II, and DVM-III. The results from all three schemes show good agreement with each other, indicating that these multiscale approaches can provide reasonable results across different flow regimes. To further analyze the convergence behavior, Figure 4 presents a comparison of the convergence history. Similar to the first test case, DVM-III demonstrates the fastest convergence among the three schemes. This can be attributed to its ability to provide more accurate predictions of the equilibrium state in the discretization of the collision term. As a result, DVM-III achieves approximately 1–2 orders of magnitude acceleration compared to DVM-I and about one order of magnitude acceleration compared to DVM-II, as shown in Table 2.

### 4.3. Case 3: Force-Driven Poiseuille Flow

This test case involves the flow of a fluid between two infinite parallel plates separated by a distance of $L_{0}=1$ in the *y*-direction. The plates maintain a temperature of $T_{0}=1$ at the reference temperature. In the force-driven Poiseuille flow, an external force is imposed in the *x*-direction. Consequently, the Boltzmann-BGK equation is modified as follows:

(45) $$\frac{\partial f_{\alpha }}{\partial t}+\xi_{\alpha }\cdot \nabla f_{\alpha }=\frac{g_{\alpha }-f_{\alpha }}{\tau }+F_{\alpha ,x}$$

where $F_{\alpha ,x}$ denotes the force term. In the context of the Maxwellian distribution function, $F_{\alpha ,x}$ can be expressed as:

(46) $$F_{\alpha ,x}=-G\frac{\partial g_{\alpha }}{\partial \xi_{\alpha ,x}}=G\frac{\xi_{\alpha ,x}-u_{x}}{R_{g}T}g_{\alpha }$$

Here, $\xi_{\alpha ,x}$ stands for the *x*-component of the discrete molecular velocity, $u_{x}$ denotes the *x*-component of the mean flow velocity, and G represents the magnitude of the external acceleration. With the external force, the corresponding modified macroscopic governing equation is as follows:

(47) $$\frac{\partial W}{\partial t}+\nabla \cdot F=S$$

where $S={\left(0,\;G,\;0,\;uG\right)}^{T}$ is the source term.

In this test example, the Knudsen number is calculated by Equation (43) with the reference density of $\rho_{0}=1$ and the dynamic viscosity is determined using Equation (44) with the viscosity index w=0.5. The apparent gas permeability is introduced to quantify the simulation results, which is defined as:

(48) $$\kappa =\frac{Kn}{\sqrt{\pi }GL_{0}^{2}}\int_{0}^{L_{0}}udy$$

In the simulation, we consider Knudsen numbers ranging from *Kn* = 10^−4^ to 10. The magnitude of the external acceleration is set as follows: $G=10^{-5}$ for the cases of 10^−4^ ≤ *Kn* < 10^−3^, $G=10^{-4}$ for the cases of 10^−3^ ≤ *Kn* < 10^−2^, $G=10^{-3}$ for the cases 10^−2^ ≤ *Kn* < 10^−1^, and $G=10^{-2}$ for the cases 10^−1^ ≤ *Kn*. The physical space is discretized uniformly into 40 cells in the *y*-direction and 5 cells in the *x*-direction. For the discretization of the molecular velocity space, we utilize the Gauss–Hermite quadrature with 8 × 8 mesh points and 28 × 28 mesh points for the cases of *Kn* < 10^−2^ and 10^−2^ ≤ *Kn* < 1, respectively. For the cases of 1 ≤ *Kn* ≤ 10, we employ the Newton–Cotes quadrature with 101 × 101 mesh points uniformly distributed in the range of [−4, 4] × [−4, 4].

**Figure 5:**
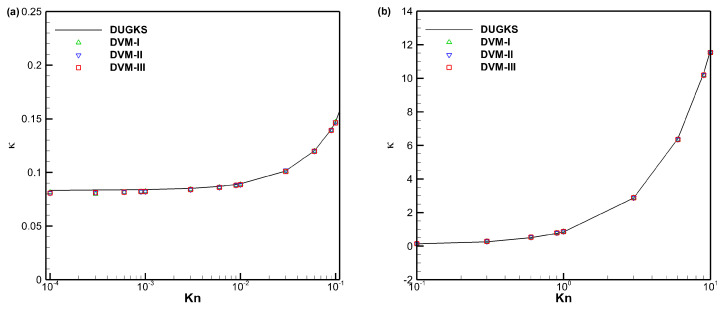
Comparison of the apparent gas permeability for force-driven Poiseuille flow with different Knudsen numbers: (**a**) 10^−4^ ≤ *Kn* ≤ 0.1, (**b**) 0.1 ≤ *Kn* ≤ 10.

**Figure 6:**
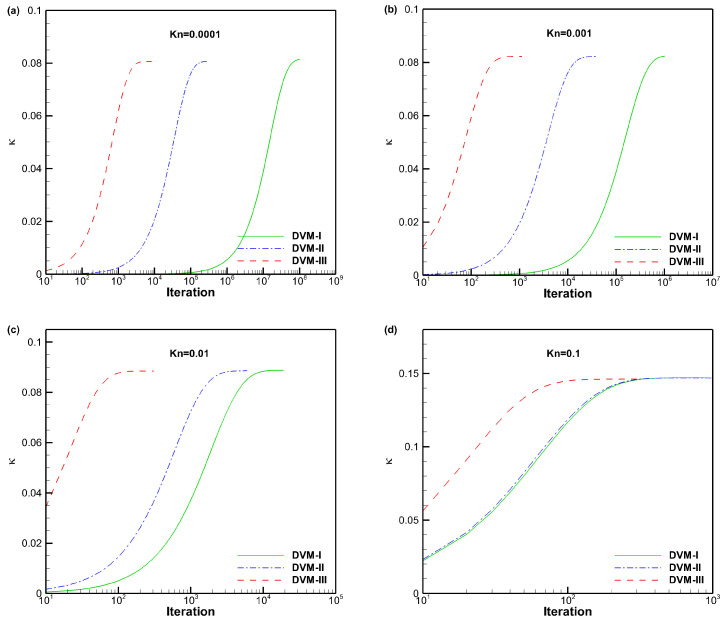
Convergence history of the apparent gas permeability for force-driven Poiseuille flow with different Knudsen numbers (**a**–**d**).

Figure 5 presents a comparison of the apparent gas permeability for force-driven Poiseuille flow with different Knudsen numbers using DVM-I, DVM-II, DVM-III, and DUGKS [41] as a reference. The results demonstrate that all three schemes yield consistent results with the reference data, indicating their accuracy as multiscale approaches. To evaluate the computational efficiency of the schemes, we analyze the convergence history of the apparent gas permeability for *Kn* = 0.0001, 0.001, 0.01, and 0.1, as shown in Figure 6. It is evident that DVM-III exhibits faster convergence compared to DVM-I and DVM-II. Specifically, for the case of *Kn* = 0.0001, DVM-III achieves approximately five and two orders of magnitude faster convergence compared to DVM-I and DVM-II, respectively. Even in the slip flow regime (*Kn* = 0.01), DVM-III still demonstrates an approximate two orders of magnitude faster convergence compared to DVM-II. These findings confirm that a more accurate prediction of the equilibrium state in the collision term discretization leads to a faster convergence rate in DVM calculations.

### 4.4. Case 4: Lid-Driven Cavity Flow

In this subsection, we will simulate the two-dimensional lid-driven cavity flow, which introduces a non-trivial dimension compared to the previous test examples. The square cavity with an edge length of $L_{0}=1$ remains stationary, except for the top wall that moves with a velocity of $u_{W}=0.15$. All walls are maintained at a fixed temperature equal to the reference temperature of $T_{0}=1$. The simulation considers three different Knudsen numbers: *Kn* = 0.075, 1 and 10, as well as two different Reynolds numbers: *Re* = 100 and 1000. For the cases with *Kn* = 0.075, 1, and 10, the dynamic viscosity is computed using Equation (44) with the viscosity index w=0.81 and the reference dynamic viscosity $\mu_{0}$ is determined by the Knudsen number:

(49) $$Kn=\frac{16\mu_{0}}{5\rho_{0}{\left(2\pi R_{g}T_{0}\right)}^{1/2}L_{0}}$$

For the cases with *Re* = 100 and 1000, the dynamic viscosity μ is directly calculated by the Reynolds number:

(50) $$\mu =\frac{\rho_{0}u_{W}L_{0}}{Re}$$

Additionally, the computational domain is uniformly discretized into 50×50 cells for the cases with *Kn* = 0.075, 1 and 10, while it is divided into 150×150 cells for the cases with *Re* = 100 and 1000. Regarding the discretization of the molecular velocity space, the Newton–Cotes quadrature with 101×101 points uniformly distributed in the range of [−4, 4]×[−4, 4] is employed for the test cases with *Kn* = 1 and 10. The Gauss–Hermite quadrature with 28×28 points is utilized for the test case with *Kn* = 0.075, and the Gauss–Hermite quadrature with 8×8 points is used for the test cases with *Re* = 100 and 1000.

**Figure 7:**
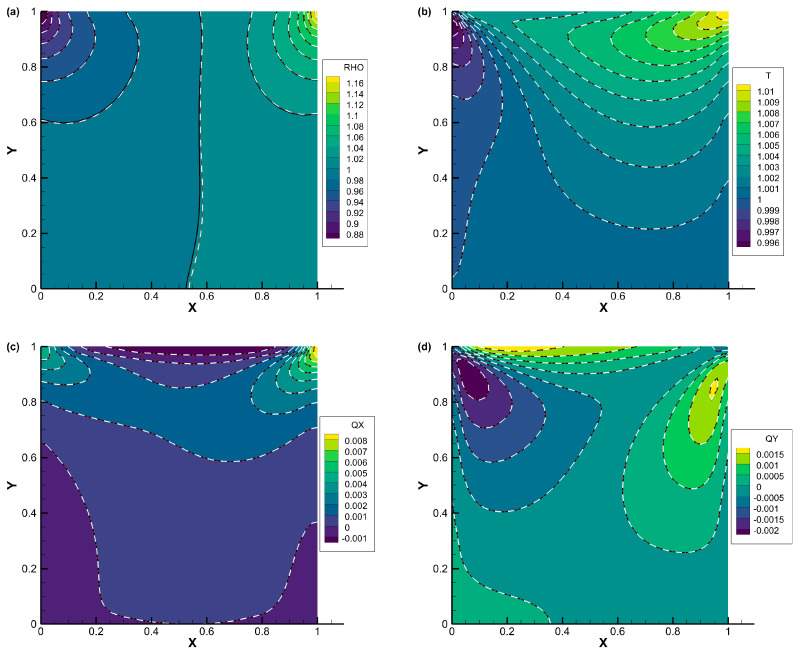
Comparison of (**a**) density, (**b**) temperature, (**c**) *x*-component of heat flux, and (**d**) *y*-component of heat flux contours for lid-driven cavity flow with *Kn* = 0.075 (DVM-I: white dash line; DVM-II: red dash-dot line; and DVM-III: colored background).

**Figure 8:**
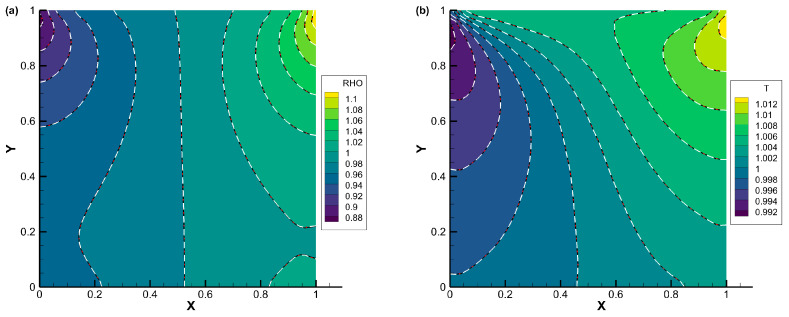

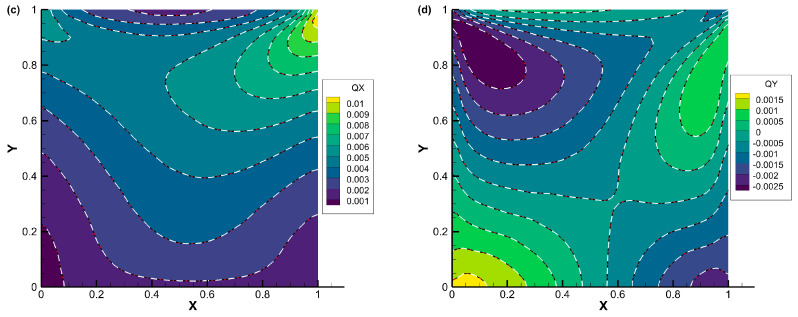
Comparison of (**a**) density, (**b**) temperature, (**c**) *x*-component of heat flux, and (**d**) *y*-component of heat flux contours for lid-driven cavity flow with *Kn* = 1 (DVM-I: white dash line; DVM-II: red dash-dot line; and DVM-III: colored background).

**Figure 9:**
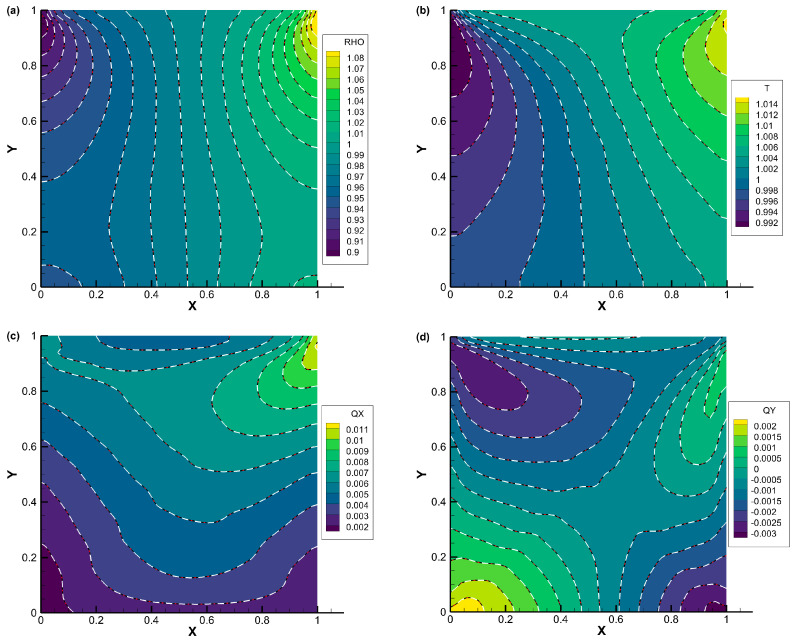
Comparison of (**a**) density, (**b**) temperature, (**c**) *x*-component of heat flux, and (**d**) *y*-component of heat flux contours for lid-driven cavity flow with *Kn* = 10 (DVM-I: white dash line; DVM-II: red dash-dot line; and DVM-III: colored background).

**Figure 10:**
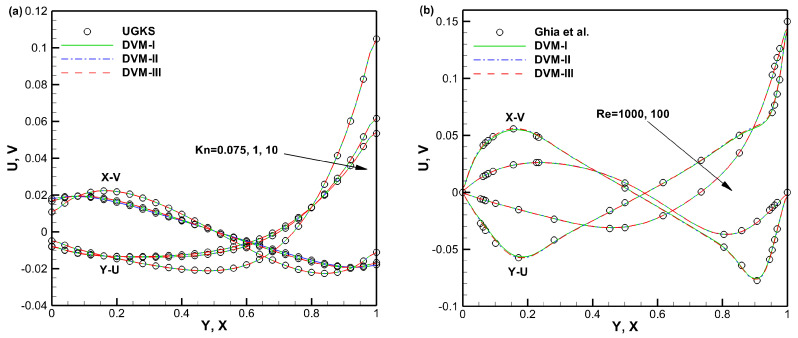
Velocity profiles along the vertical and horizontal central lines of the cavity with different Knudsen (**a**)/Reynolds (**b**) numbers [43].

**Figure 11:**
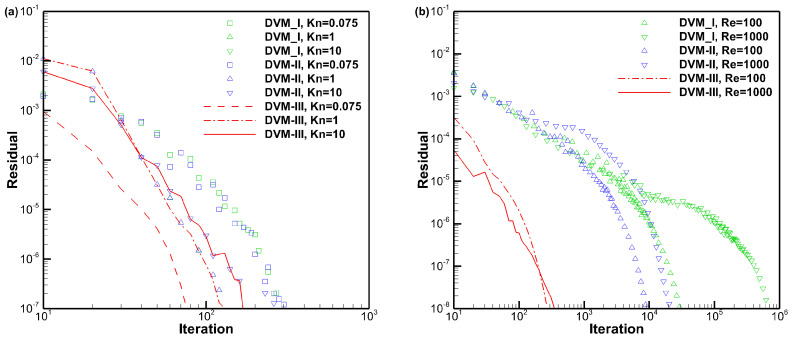
Convergence history for lid-driven cavity flow with different Knudsen (**a**)/Reynolds (**b**) numbers.

The comparisons of density, temperature, the *x*-component of heat flux, and the *y*-component of heat flux contours for lid-driven cavity flow with Knudsen numbers of 0.075, 1, and 10 are displayed in Figure 7, Figure 8 and Figure 9, respectively. The results obtained from all three schemes (DVM-I, DVM-II, and DVM-III) exhibit good agreement, indicating their capability to accurately capture the flow behavior across different regimes. Figure 10 provides a quantitative comparison of the velocity profiles along the vertical and horizontal central lines of the cavity for the three schemes, along with the results of UGKS [42] and the numerical results of Ghia et al. [43] as reference data. Once again, the results obtained from all three schemes closely align with the reference data, further supporting the applicability of these multiscale approaches in the different flow regimes. To assess the convergence performance, Figure 11 compares the convergence history for lid-driven cavity flow with different Knudsen/Reynolds numbers. Additionally, Table 3 quantitatively compares the computational cost among the three schemes. Clearly, DVM-III exhibits the fastest convergence rate among the three schemes, achieving approximately 1–2 orders of magnitude faster convergence compared to DVM-I. This observation confirms that the accuracy in predicting the equilibrium state for the collision term’s discretization plays a critical role in accelerating convergence in DVM calculations.

### 4.5. Case 5: Flow around a NACA0012 Airfoil

This test case involves the flow around a NACA0012 airfoil with a Mach number of *Ma* = 0.2 and an angle of attack of *AoA* = 10 degrees. The temperature of the airfoil is maintained at the reference temperature of $T_{0}=1$. The Knudsen number is defined by Equation (43) using the reference density of $\rho_{0}=1$ and the normalized airfoil chord length of $L_{0}=1$, and the dynamic viscosity is determined using Equation (44) with the viscosity index w=0.81. Consequently, the relationship between the Knudsen number, Mach number, and Reynolds number can be expressed as:

(51) $$Kn=\frac{Ma}{Re}\sqrt{\frac{\gamma \pi }{2}}$$

In the simulation, we consider three different Reynolds numbers: *Re* = 5, 50, and 500. The computational domain extends up to a distance of $25L_{0}$ from the center of the airfoil and is discretized using an unstructured mesh comprising 16,042 triangular cells. On the airfoil surface, we place 200 discrete points. For the discretization of the molecular velocity space, we employ the Gauss–Hermite quadrature with a mesh of 28 × 28 mesh points.

**Figure 12:**
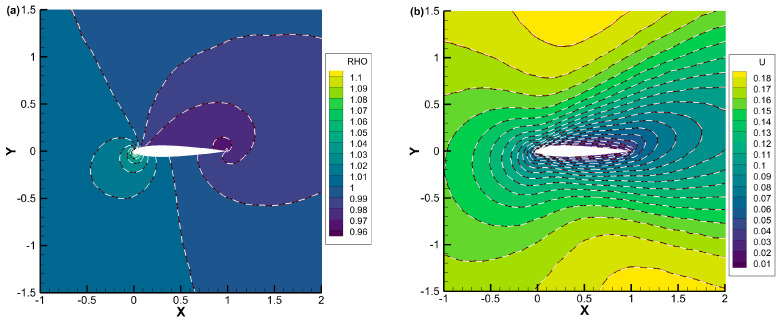

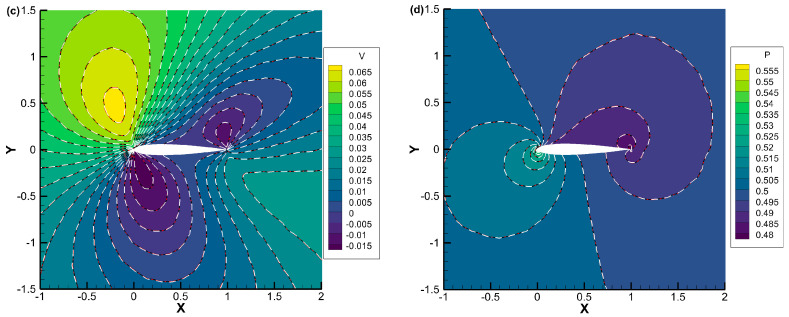
Comparison of (**a**) density, (**b**) *u*-velocity, (**c**) *v*-velocity, and (**d**) pressure contours for flow around a NACA0012 airfoil with *Re* = 5 (DVM-I: white dash line; DVM-II: red dash-dot line; and DVM-III: colored background).

**Figure 13:**
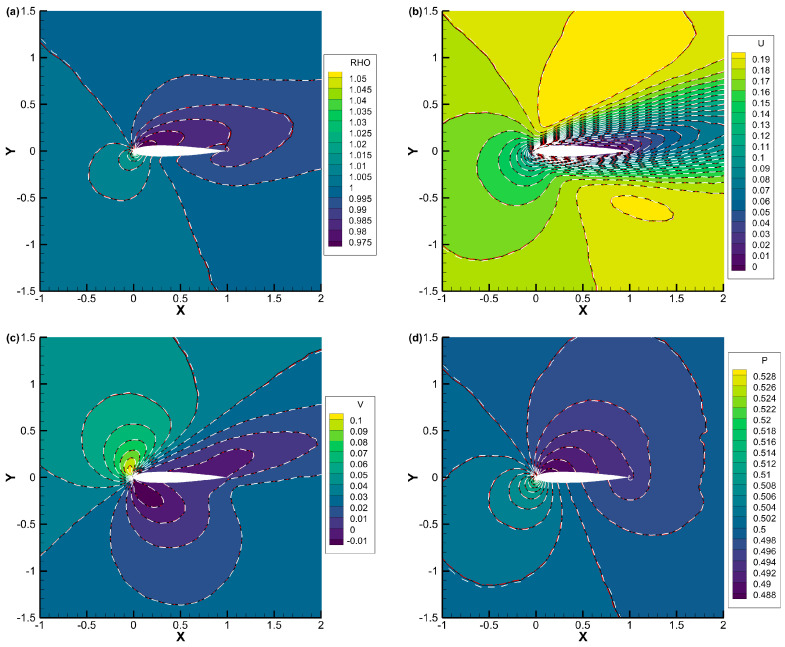
Comparison of (**a**) density, (**b**) *u*-velocity, (**c**) *v*-velocity, and (**d**) pressure contours for flow around a NACA0012 airfoil with *Re* = 50 (DVM-I: white dash line; DVM-II: red dash-dot line; and DVM-III: colored background).

**Figure 14:**
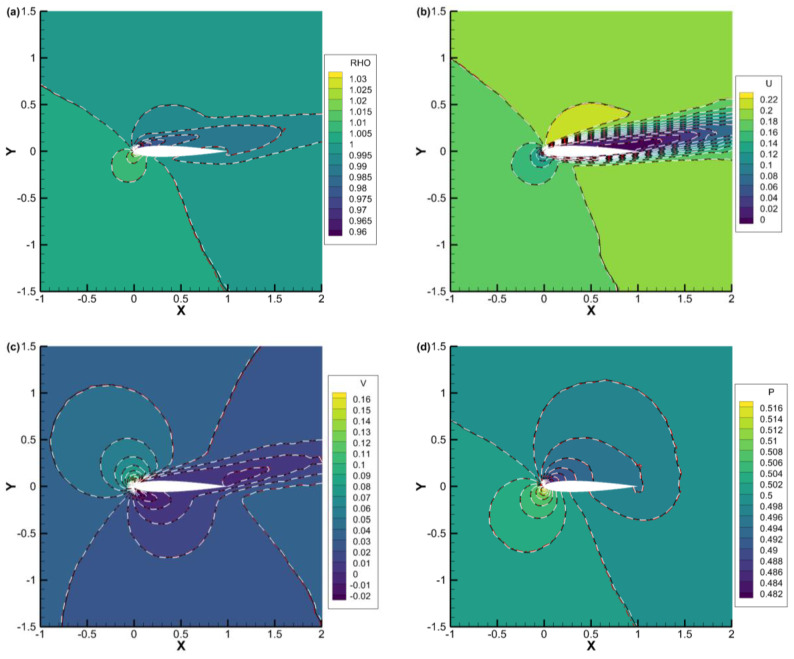
Comparison of (**a**) density, (**b**) *u*-velocity, (**c**) *v*-velocity, and (**d**) pressure contours for flow around a NACA0012 airfoil with *Re* = 500 (DVM-I: white dash line; DVM-II: red dash-dot line; and DVM-III: colored background).

**Figure 15:**
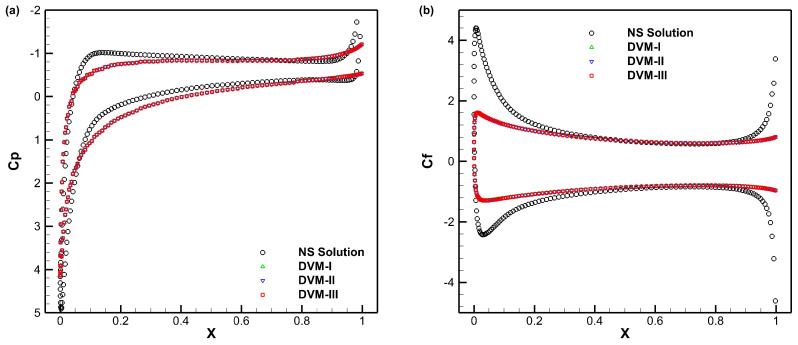
Comparison of pressure coefficient (**a**) and skin friction coefficient (**b**) distributions along the airfoil surface for flow around a NACA0012 airfoil with *Re* = 5.

**Figure 16:**
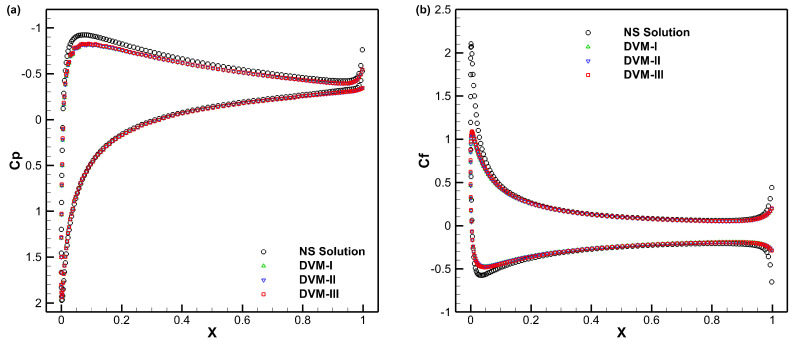
Comparison of pressure coefficient (**a**) and skin friction coefficient (**b**) distributions along the airfoil surface for flow around a NACA0012 airfoil with *Re* = 50.

**Figure 17:**
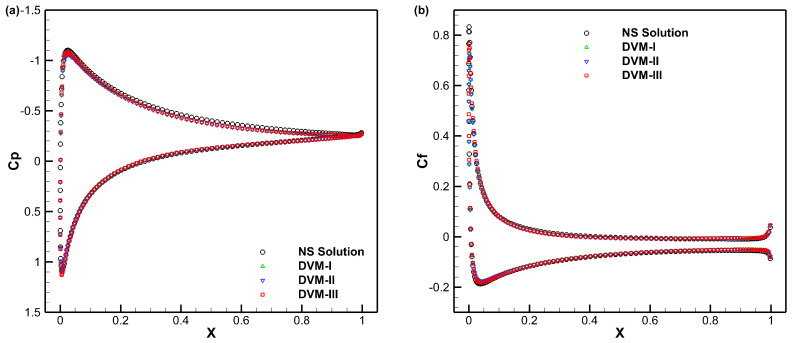
Comparison of pressure coefficient (**a**) and skin friction coefficient (**b**) distributions along the airfoil surface for flow around a NACA0012 airfoil with *Re* = 500.

**Figure 18:**
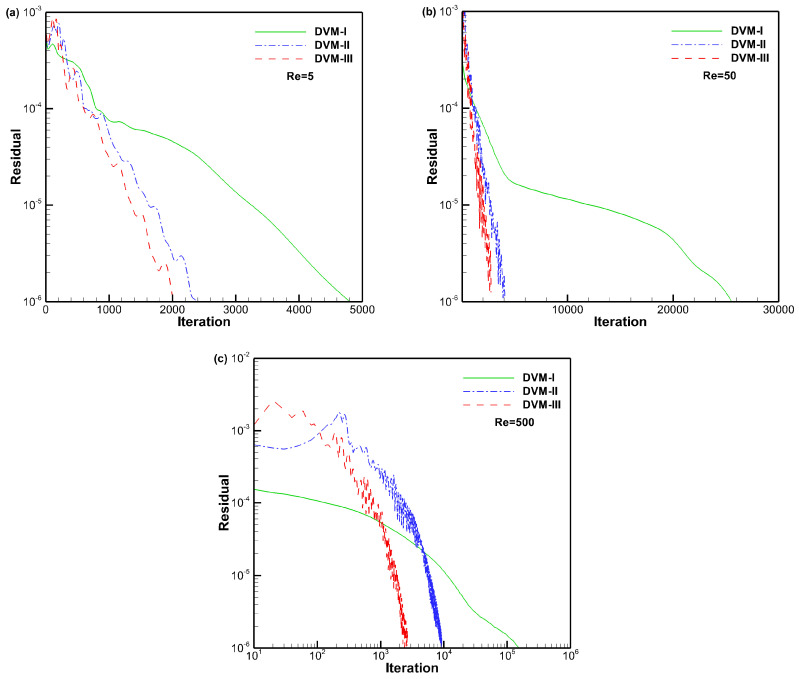
Convergence history for flow around a NACA0012 airfoil with different Reynolds numbers (**a**–**c**).

Figure 12, Figure 13 and Figure 14 present the density, *u*-velocity, *v*-velocity, and pressure contours for flows around a NACA0012 airfoil at Reynolds numbers of 5, 50, and 500, respectively. The results obtained by the DVM-I, DVM-II, and DVM-III are found to be consistent with each other. Figure 15, Figure 16 and Figure 17 compare the pressure coefficient and skin friction coefficient distributions along the airfoil surface for the cases of *Re* = 5, 50, and 500. These figures also include numerical results obtained using the Navier-Stokes equation with a conventional CFD scheme [44]. It is observed that the results obtained by DVM-I, DVM-II, and DVM-III align well with each other and show good agreement with the results from the Navier–Stokes equation for the case of *Re* = 500. However, for the cases of *Re* = 5 and 50, there are deviations between the results obtained by the three schemes and those from the Navier–Stokes equation due to the rarefaction effect. In Figure 18, we compare the convergence history of the three schemes at different Reynolds numbers. It is evident that DVM-III exhibits faster convergence compared to the other two schemes. Specifically, for the case of *Re* = 500, there is an acceleration of approximately one order of magnitude compared to DVM-I.

### 4.6. Case 6: Flow in a Planar Microchannel

**Figure 19:**
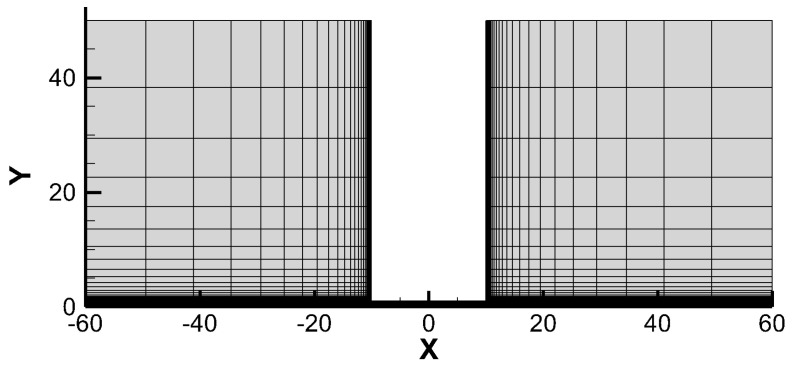
Geometry and computational mesh in the physical space for flow in a planar microchannel.

The last test example focuses on the flow in a planar microchannel, which has been previously investigated by Titarev [45,46]. The configuration of this test case is illustrated in Figure 19 and consists of a left reservoir with pressure $p_{1}$ and temperature $T_{1}$, a right reservoir with pressure $p_{2}$ and temperature $T_{2}$, and a channel with the aspect ratio of l/a=10, where l represents half of the length and a represents half of the width. In our simulation, $p_{1}/p_{2}=1.1$ and $T_{1}/T_{2}=1$ are considered. To compare our results with those obtained by Titarev [45,46], we calculate the collision time using τ=1/δ, where δ is a rarefaction parameter that is inversely proportional to the Knudsen number. In the simulation, we examine six different values of δ: 0.01, 0.1, 1, 10, 100, and 1000. Note that δ=1000 corresponds to the continuum flow regime, which poses a challenge for DVM-I to efficiently achieve convergence and was not tested in the work of Titarev [45,46].

To quantitatively compare the simulation results with the reference data provided by Titarev [45,46], the non-dimensional mass flow rate is introduced

(52) $$Mp=-\frac{2l}{p_{2}-p_{1}}M,$$

with:

(53) $$M=\frac{\sqrt{2R_{g}T_{0}}}{ap_{0}}\int_{0}^{a}\rho \left(0,y\right)u\left(0,y\right)dy,$$

where $p_{0}=\left(p_{1}+p_{2}\right)/2$ and $T_{0}=\left(T_{1}+T_{2}\right)/2$ represent the reference pressure and reference temperature, respectively. In the simulation, the physical space is discretized by 5501 quadrilateral cells, and the molecular velocity space is discretized by the Gauss–Hermite quadrature with a mesh of 28 × 28 mesh points for δ≤100 and 8 × 8 mesh points for δ≤1000.

**Figure 20:**
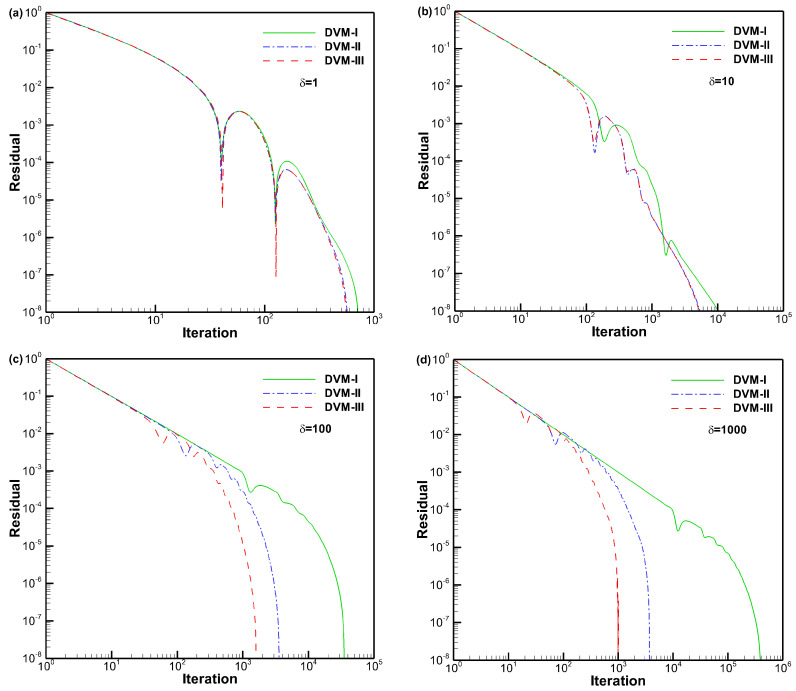
Convergence history for flow in a planar microchannel with different rarefaction parameters (**a**–**d**).

Figure 20 illustrates the convergence history for the flow in a planar microchannel with different rarefaction parameters. In this figure, we only display the convergence history obtained by DVM-I, DVM-II, and DVM-III for the cases of δ≥1, since the convergence rates of the three schemes are identical for the cases of δ=0.01 and 0.1. It is evident that DVM-III exhibits the fastest convergence, followed by DVM-II, while DVM-I converges at a slower rate. Particularly for the case of δ=1000, both DVM-II and DVM-III achieve a significant acceleration of convergence by two orders of magnitude compared to DVM-I. Furthermore, Table 4 presents the non-dimensional mass flow rates obtained by the three schemes, alongside the reference data provided by Titarev [46]. Basically, the simulation results demonstrate good agreement with the reference data, validating the accuracy of all three schemes.

## 5. Conclusions

In this study, three versions of multiscale DVM are compared to investigate the key factor in accelerating convergence in DVM calculations. To ensure accuracy across different flow regimes, particularly in the near-continuum and continuum flows, these approaches use the local discrete characteristic solution of the Boltzmann-BGK equation to calculate the numerical flux at the cell interface, similar to the DUGKS. The first version, DVM-I, employs a semi-implicit scheme to discretize the collision term. It approximates the equilibrium state using its current time step value. On the other hand, DVM-II and DVM-III utilize a fully implicit scheme to discretize the Boltzmann-BGK equation, including the collision term. In these versions, the equilibrium state is predicted based on the solution of the corresponding macroscopic governing equation. Notably, DVM-III introduces an inner iteration of the macroscopic governing equation between adjacent DVM steps, leading to a more accurate prediction of the equilibrium state for the fully implicit discretization of the collision term.

To investigate the key factor in accelerating convergence in DVM calculations, simulations have been conducted on six benchmark cases, including Couette flow, heat transfer between two parallel plates, force-driven Poiseuille flow, lid-driven cavity flow, flow around a NACA0012 airfoil, and flow in a planar microchannel. By considering the collisional effect in the calculation of the numerical flux at the cell interface, all three multiscale approaches (DVM-I, DVM-II, and DVM-III) yield reasonable results in good agreement. Concerning computational efficiency, DVM-III exhibits the highest efficiency in near-continuum and continuum flow regimes, followed by DVM-II, while DVM-I shows a lower efficiency. This indicates that the fully implicit discretization of the collision term plays a crucial role in accelerating convergence in DVM computations. Additionally, the more precise prediction of the equilibrium state resulted in a higher convergence rate. These findings provide valuable insights into the development of efficient and accurate multiscale approaches for simulating flows around irregular objects in near-space environments [47,48]. However, it is crucial to acknowledge that for practical applications, parallel implementation becomes necessary due to the substantial computational cost associated with the DVM. Employing multicore calculations alongside the LU-SGS scheme can significantly impact convergence. Furthermore, implementing parallelization for DVM-II and DVM-III, where resolving the macroscopic governing equation is required, poses greater challenges compared to DVM-I. Developing parallel versions for these schemes will be addressed in future work.

## Figures and Tables

**Table 1:** Computational cost (hours) of DVM-I, DVM-II, and DVM-III for Couette flow with different Knudsen numbers.

*Kn*	0.001	0.01	0.1	1	10
DVM-I	13.120	0.396	0.0269	0.208	0.391
DVM-II	1.404	0.274	0.0225	0.219	0.412
DVM-III	0.0608	0.0258	0.0237	0.214	0.409
Ratio 1	9.34	1.44	1.20	0.95	0.95
Ratio 2	215.79	15.35	1.14	0.97	0.96

Note: “Ratio 1” represents the speed-up ratio of DVM-II over DVM-I, while “Ratio 2” represents the speed-up ratio of DVM-III over DVM-I.

**Table 2:** Computational cost (hours) of DVM-I, DVM-II, and DVM-III for heat transfer between two parallel plates with different Knudsen numbers.

*Kn*	0.001	0.01	0.1	1	10
DVM-I	8.550	0.320	0.0224	0.160	0.314
DVM-II	0.903	0.182	0.0237	0.169	0.331
DVM-III	0.0323	0.0269	0.0248	0.164	0.333
Ratio 1	9.47	1.76	0.95	0.95	0.95
Ratio 2	264.71	11.90	0.90	0.98	0.94

Note: “Ratio 1” and “Ratio 2” have the same definitions as provided in Table 1.

**Table 3:** Computational cost (hours) of the DVM-I, DVM-II, and DVM-III for lid-driven cavity flow with different Knudsen/Reynolds numbers.

*Re*/*Kn*	1000	100	0.075	1	10
DVM-I	275.478	12.109	0.0705	0.498	0.613
DVM-II	9.102	3.873	0.0766	0.526	0.648
DVM-III	0.370	0.285	0.0234	0.541	0.666
Ratio 1	30.27	3.127	0.92	0.95	0.95
Ratio 2	744.53	42.488	3.01	0.92	0.92

Note: “Ratio 1” and “Ratio 2” have the same definitions as provided in Table 1.

**Table 4:** Mass flow rate for flow in a planar microchannel with different rarefaction parameters.

*δ*	0.01	0.1	1	10	100	1000
DVM-I	2.69	2.58	2.79	8.01	48.1	88.4
DVM-II	2.69	2.58	2.79	8.01	47.7	88.2
DVM-III	2.69	2.59	2.80	8.12	48.6	88.3
Titarev [46]	2.69	2.58	2.77	7.99	47.8	-

## Data Availability

Data is contained within the article.
